# Ibrutinib inhibits SDF1/CXCR4 mediated migration in AML

**DOI:** 10.18632/oncotarget.2479

**Published:** 2014-09-16

**Authors:** Lyubov Zaitseva, Megan Y. Murray, Manar S. Shafat, Matthew J. Lawes, David J. MacEwan, Kristian M. Bowles, Stuart A. Rushworth

**Affiliations:** ^1^ Department of Molecular Haematology, Norwich Medical School, University of East Anglia, Norwich Research Park, Norwich, United Kingdom; ^2^ Department of Molecular and Clinical Pharmacology, Institute of Translational Medicine, University of Liverpool, Liverpool, United Kingdom; ^3^ Department of Haematology, Norfolk and Norwich University Hospitals NHS Trust, Colney Lane, Norwich United Kingdom

**Keywords:** AML, BTK, Ibrutinib, CXCR4, SDF1

## Abstract

Pharmacological targeting of BTK using ibrutinib has recently shown encouraging clinical activity in a range of lymphoid malignancies. Recently we reported that ibrutinib inhibits human acute myeloid leukemia (AML) blast proliferation and leukemic cell adhesion to the surrounding bone marrow stroma cells. Here we report that in human AML ibrutinib, in addition, functions to inhibit SDF1/CXCR4-mediated AML migration at concentrations achievable *in vivo*. It has previously been shown that SDF1/CXCR4-induced migration is dependent on activation of downstream BTK in chronic lymphocytic leukaemia (CLL) and multiple myeloma. Here we show that SDF-1 induces BTK phosphorylation and downstream MAPK signalling in primary AML blast. Furthermore, we show that ibrutinib can inhibit SDF1-induced AKT and MAPK activation. These results reported here provide a molecular mechanistic rationale for clinically evaluating BTK inhibition in AML patients and suggests that in some AML patients the blasts count may initially rise in response to ibrutinib therapy, analgous to similar clinical observations in CLL.

## INTRODUCTION

Despite recent significant progress in the understanding of the biology of AML the clinical outcomes for the majority of patients diagnosed with AML presently remain poor. AML is primarily a disease of the elderly[1], and for most of the 75% of patients aged over 60 at diagnosis long term survival has barely improved in the last 40 years [2], largely because the intensity and side effects of existing curative therapeutic strategies (which are commonly used to treat the younger fitter patients) coupled to patient co-morbidities, frequently limit their use in this older less fit population. Consequently, there is an urgent need to identify pharmacological strategies in AML, which are not only effective but can be tolerated by the older, less well patient. It is envisaged that treatments which target tumor-specific biology will help realise this goal.

Bruton’s Tyrosine Kinase (BTK) is a non-receptor tyrosine kinase that belongs to the Tec family and clearly has an important function in a number of benign and malignant cells of the haematopoietic system [3-7]. Moreover, recent phase 1, 2 and 3 studies of the irreversible oral BTK inhibitor, ibrutinib have demonstrated excellent clinical activity and tolerability against a variety of B-cell malignancies including, chronic lymphocytic leukemia (CLL), mantle cell lymphoma, hairy cell leukaemia and diffuse large B-cell lymphoma in younger and older patients alike [8-16]. Furthermore, it is now clear that the mechanism of action [MOA] of ibrutinib is multifactorial in nature with a significant component of its function in lymphoid malignancy involving disruption of the interaction between the tumor cell and the microenvironment that protects it. Recently our group and others have shown that there is high BTK phosphorylation and RNA expression in AML [17-19]. Moreover, our recent study described for the first time that ibrutinib and BTK-targeted RNA interference reduced factor-induced proliferation of both AML cell lines and primary AML blasts, as well as reducing AML blast adhesion to BMSC [19].

Inhibition of BTK has been shown to regulate CLL, MCL and MM cell migration by inhibiting SDF1 (stromal derived factor 1) induced CXCR4 regulated cell trafficking [20-22]. SDF1 is an extracellular chemokine which is abundantly produced by the bone marrow microenvironment binds to and activates its receptor CXCR4 which is highly expressed on many non-malignant and malignant cells including breast cancer cell, normal haematopoietic CD34+ cells as well as AML blasts [23, 24]. In this study we investigate the role of BTK inhibition on the function of SDF-1/CXCR4 in AML. Furthermore, we examine the intracellular signalling cascade down stream of SDF1/CXCR4 activation, specifically the effects on AKT and MAPK. Finally, we examine the role of BTK RNAi on SDF-1/CXCR4-mediated AML migration.

## RESULTS

### Ibrutinib inhibits AML cell migration in response to SDF-1

**Figure 1:**
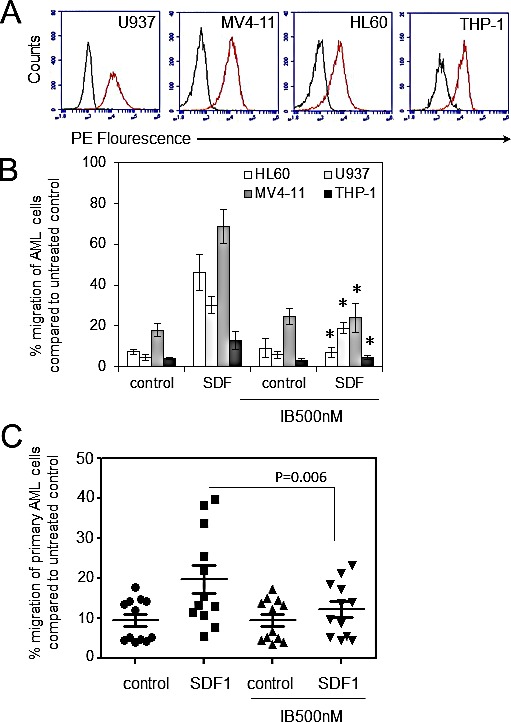
Ibrutinib inhibits AML cell migration in response to SDF-1 (A) AML cell lines were examined for CXCR4 expression (n=4) using flow cytometry (B) AML cell lines were pretreated with 500nM of ibrutinib for 1 h before wash off and then placed in the upper well of a 8.0μM transwell plate. The lower chamber contained 500ul of serum free media supplemented with SDF1 (100 ng/ml) for 3 hours and then assessed for cell number using a flow cytometer. Data were normalised to DMSO treated cells. *statistical significance. (C) Primary AML blasts (n=12) were pretreated with ibrutinib (500 nM) for 1 h before wash off and then placed in the upper well of a 8.0μM transwell plate. The lower chamber contained 500ul of serum free media supplemented with SDF1 (100 ng/ml) for 3 hours and then assessed for cell number using a flow cytometer. Data were normalised to DMSO treated cells.

Previously we have shown that ibrutinib inhibits AML proliferation and adhesion to BMSC. In myeloma and CLL ibrutinib inhibits CXCR4/SDF1-mediated migration [11, 25]. Moreover, inhibition of CXCR4/SDF1 axis using AMD3100 (Plerixafor), an agonist for CXCR4, has been shown to decrease AML tumor burden in AML leukaemic mice in combination with conventional AML chemotherapy [26]. Therefore we initially examined the expression of CXCR4 in human AML cell lines and found that 4/4 cell lines were positive for CXCR4 expression (Figure 1A). Next we examined the effects of ibrutinib on the migration of the AML cell lines U937, MV4-11, HL60 and THP-1 in response to SDF1. Figure 1B shows that ibrutinib can inhibit the migration of all AML cell lines tested. Finally, we tested the in-vitro activity of ibrutinib on SDF-1 induced migration in a spectrum of primary AML blasts from a wide age spectrum of adult patients and across a range of WHO AML subclasses (Table 1). Figure 1C shows that ibrutinib significantly inhibits primary AML blast migration (n=12). Taken together these data show that ibrutinib inhibits SDF1/CXCR4 driven migration in human AML.

**Table 1:** AML patient sample information This table defines the nature of the AML disease including WHO diagnosis and cytogenetics.

Number	Age	Gender	WHO diagnosis	Cytogenetics	%Blasts
AML#1	82	M	AML NOS	not available	65%
AML#2	47	M	AML without maturation	not available	90%
AML#3	55	F	Acute monoblastic and monocytic leukaemia	normal	80%
AML#4	41	F	AML with t(6:9)(p23:q34);DEK-NUP2I4	t(6:9)	>95%
AML#5	77	F	AML with maturation	normal	65%
AML#6	70	M	AML with minimal differentiation	normal	>95%
AML#7	40	M	AML with minimal differentiation	normal	90%
AML#8	70	M	AML without maturation	complex	95%
AML#9	91	F	AML NOS	not available	60%
AML#10	55	F	AML relapsed	not available	70%
AML#11	59	F	AML with t(8:21)(q22:q22);RUNX1-RUNX1T1	t(8;21)	80%
AML#12	51	M	AML with monocytic differentiation	normal	95%

### BTK is activated in response to SDF-1 in human AML

**Figure 2:**
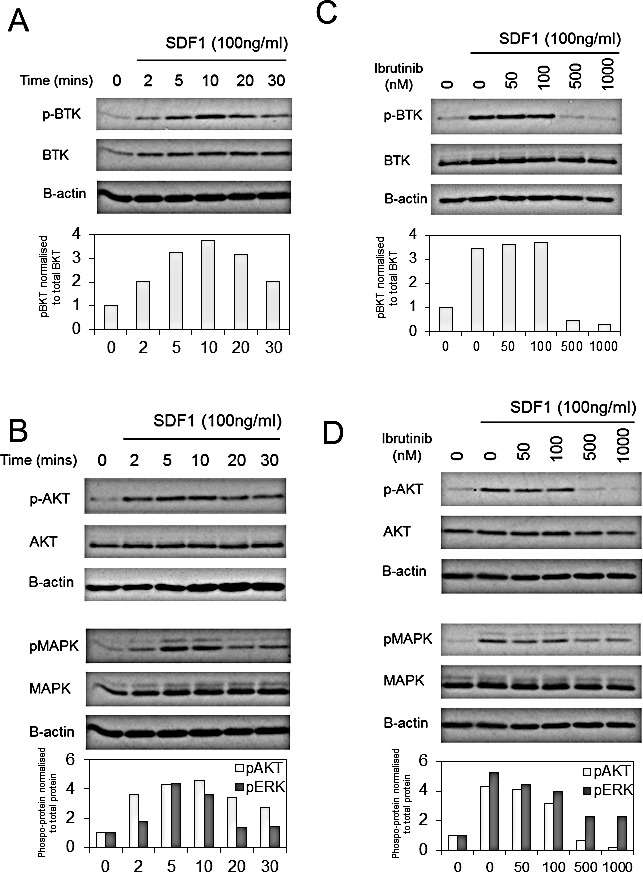
BTK is activated in response to SDF-1 in human AML (A) AML blasts were treated with SDF1 (100 ng/ml) for indicated times. Protein extracts were obtained and Western blot analysis was conducted for pBTK, BTK, pMAPK, MAPK and β-actin protein levels. (B) AML blasts were pretreated with increasing concentrations of ibrutinib for 1h and then treated with SDF1 (100 ng/ml) for 10 mins. Protein extracts were obtained and Western blot analysis was conducted for pATK, ATK, pMAPK, MAPK and β-actin protein levels. These results are representative of experiments which were repeated three times with the bar graphs showing mean expression (as measured by densitometry) compared to control from all 3 experiments.

Since SDF1 has been shown to mediate migration of AML in a CXCR4 dependent mechanism and that SDF1/CXCR4-induced migration is dependent on activation of downstream BTK in CLL, myeloma and normal B cells [21, 27], we examined the activity of BTK phosphorylation and downstream AKT and MAPK in response to SDF1 and found that SDF1 increases levels of pBTK and downstream MAPK in primary AML blasts (Figure 2A). Since CXCR4 activation has been shown to mediate downstream MAPK and AKT activity [28, 29], we examined if ibrutinib could inhibit SDF1 induced MAPK and AKT signalling in primary AML blast. Figure 2B shows that ibrutinib inhibits SDF1 induced BTK, MAPK and AKT phosphorylation in AML at concentrations equivalent to those that can be achieved *in vivo* (based on ibrutinib 420 mg orally once a day) [10, 16]. These results demonstrate that ibrutinib inhibits CXCR4 mediated signalling in primary human AML cells.

### Pharmacological inhibition of G proteins inhibits SDF1 induced BTK activation

**Figure 3:**
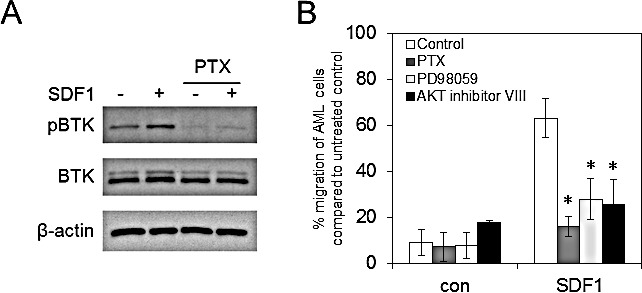
Pharmacological inhibition of G-proteins, AKT and ERK block SDF1 induced migration in AML (A) MV4-11 cells were pretreated with pertussis toxin (100ng/ml) for 30 mins and then stimulated with SDF1 for 10mins. Protein extracts were obtained and Western blot analysis was conducted for pBTK, BTK and β-actin protein levels. These results are representative of experiments which were repeated three times. (B) MV4-11 cells were pretreated with pertussis toxin (100ng/ml), PD98059 (20 μM) and AKT inhibitor VIII (2 μM) for 30 mins and then placed in the upper well of a 8.0μM transwell plate. The lower chamber contained 500ul of serum free media supplemented with SDF1 (100 ng/ml) for 3 hours and then assessed for cell number using a flow cytometer. Data were normalised to DMSO treated cells. *statistical significance compared to SDF1 MV4-11 cells.

Since CXCR4 is a Gi-coupled receptor and pertussis toxin inhibits Gi-coupled receptor activation [30], we used pertussis toxin to determine if SDF1 induces phosphorylation of BTK through CXCR4. To do this we treated AML cell line MV4-11 with pertussis toxin for 15 mins before the addition of SDF1 for 10mins. Figure 3A shows that pertussis toxin can inhibit SDF1 mediated phospoBTK activation in AML blasts. Next we wanted to determine if inhibition of CXCR4 activation by pertussis toxin as well as inhibition of downstream AKT and MAPK, could also block SDF1 induced migration of AML cell line MV4-11. Figure 3B shows that pertussis toxin, PD98059 and AKT inhibitor VIII could all inhibit SDF1 induced migration in MV4-11 cells.

### Knockdown of BTK inhibits SDF1 induced migration in AML

**Figure 4:**
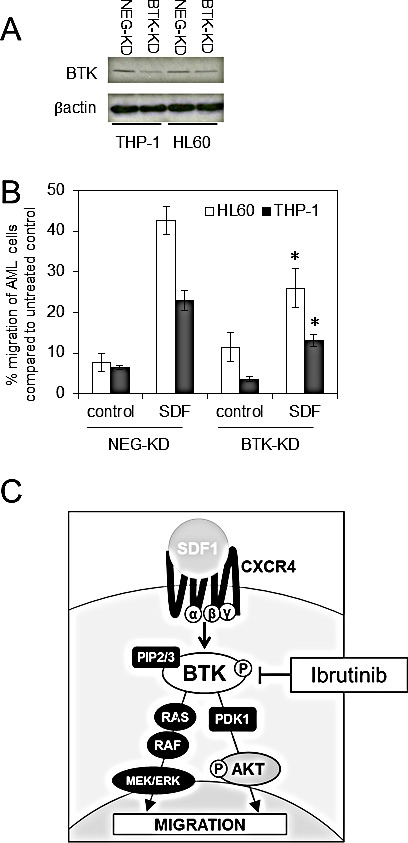
Knockdown of BTK inhibits SDF1 induced migration in AML (A) AML cell lines (HL60 and THP-1) were transduced with BTK-targeted miRNA (BTK-KD) or a negative-targeted miRNA (NEG-KD) GFP-tagged lentiviral constructs for 72 h. Protein extracts were obtained and Western blot analysis was conducted for BTK and β-actin protein levels. These results are representative of experiments which were repeated three times (B) HL60 and THP-1 were transduced with BTK-KD and NEG-KD lentivirus for 72 h and then placed in the upper well of a 8.0μM transwell plate. The lower chamber contained 500ul of serum free media supplemented with SDF1 (100 ng/ml) for 3 hours and then assessed for cell number using a flow cytometer. Data were normalised to NEG-KD cells. *statistical significance. (C) Schematic to show the role of BTK inhibition by ibrutinib in preventing AML migration.

It has been shown that ibrutinib has multiple kinase targets including interleukin-2-inducible kinase [31], as well as other members of the TEC kinase family. Therefore to eliminate the problems associated with off target inhibitor activity we evaluated migration of AML cells lines using genetic inhibition of BTK. To do this we generated lentivirus-mediated long-term BTK knockdown using targeted artificial microRNA (BTK-targeted miRNA) and visualisation of infected cells by a concurrently expressed GFP signal tag as previously described [19]. The introduction of BTK-specific miRNA dramatically inhibited the expression of BTK in THP-1 and HL60 (Figure 4A). Next we examined the role of mRNA targeted BTK knockdown on THP-1 and HL60 migration. Figure 4B shows that AML cells with BTK-KD had reduced SDF1 mediated migration confirming that BTK is involved in regulating AML migration in response to SDF1. Figure 4C shows a schematic of how BTK targets SDF1/CXCR4 signalling in AML.

## DISCUSSION

BTK is a cytoplasmic tyrosine kinase widely expressed in hematopoietic cells and long known to be critical in B cell differentiation and survival pathways. BTK is a member of the BTK/Tec family of tyrosine kinases [32]. BTK activation has been implicated in a variety of hematopoietic cellular responses and there is a growing literature supporting the role of BTK in HSC and cells of the myeloid compartment [33-35]. Previously we have shown that ibrutinib inhibits AML adhesion to BMSC. In the present study we develop on these findings to investigate the effect of BTK inhibition using ibrutinib on AML blast migration.

In CLL and other B cell malignancies ibrutinib inhibits SDF1/CXCR4-induced tumor cell migration [20, 25]. We hypothesised that SDF1/CXCR4 signalling was also regulated by BTK in AML. Inhibition of CXCR4 signalling using the CXCR4 inhibitor AMD3100 (Plerixafor) can mobilise non-malignant hematopoietic stem cells from the bone marrow in to the peripheral blood for harvesting[36]. Moreover, in AML AMD3100 has been shown to increase the level of AML cells in the peripheral blood and therefore increase their sensitivity to chemotherapy in murine models [26]. Our data confirm that ibrutinib targets SDF1/CXCR4 signalling.

Ibrutinib has been shown to be well tolerated with limited grade 3 and 4 toxicity across a broad age range of patients with CLL and MCL [5, 15, 16]. Since AMD3100 inhibits SDF1-mediated non-malignant CD34+ cell migration we also expect ibrutinib to do the same, however, as we have observed previously we expect ibrutinib to inhibit pro-survival signals including SCF mediated and to a lesser extent IL-3 and GM-CSF in AML [19]. The clinical significance of the ibrutinib effect on non-malignant HSC in patients with AML can only be defined in the context of clinical trials.

In patients with CLL treated with ibrutinib an initial and sometimes persistent rise in the circulating lymphocyte count may be seen, even in the context of clinically responding disease [8]. This is believed to occur in part because of an egress of malignant cells from nodal compartments[37]. In AML blocking of SDF/CXCR4 by AMD3100 is associated with a rise in circulating tumour cells[26]. In light of these reports and our own findings that Ibrutinib inhibits CXCR4/SDF1 mediated migration in AML we hypothesise that in some AML patients the blast count may initially rise in response to ibrutinib therapy. However while the rising lymphocyte count in CLL causes no known harm to the patient, an uncontrolled rise in the blast count in AML may put patients at risk of leucostasis. Accordingly we suggest future clinical trial protocols of BTK inhibition in human AML should include strategies to manage a rising blast count (such as addition of cytoreductive chemotherapy) should it occur.

Here we show for the first time that in human AML ibrutinib inhibits SDF1/CXCR4 pathway and functions to disrupt migration signals from the microenvironment This study further validates an emerging focus of targeting kinases critical for the survival of malignant cells and gives us a clearer understanding of how inhibition of BTK may optimally be harnessed therapeutically in AML. In summary, we provide additional scientific support for clinical trials of ibrutinib in patients with AML.

## METHODS

### Materials

Anti-phosphorylated and pan AKT, BTK and MAPK antibodies were purchased from Cell Signaling Technology (Cambridge, MA). Anti-CD34-PE and anti-CXCR4-FITC antibodies were purchased from Miltenyi Biotec (Auburn, CA). Ibrutinib was obtained from Selleck Chemicals. SDF1 was purchased from Miltenyi Biotec. Pertusis toxin was purchased from R&D systems (Abingdon, UK). LY294002 and U0126 were purchased from Cell Signalling Technology. All other reagents were obtained from Sigma-Aldrich (St Louis, MO), unless indicated.

### Cell culture

AML cells were obtained from patients’ bone marrow or blood following informed consent and under approval from the UK National Research Ethics Service (LRECref07/H0310/146). For primary cell isolation, heparinized blood was collected from volunteers and human peripheral blood mononuclear cells (PBMCs) isolated by Histopaque (Sigma-Aldrich, UK) density gradient centrifugation. AML samples that were less than 80% blasts were purified using the CD34 positive selection kit (denoted by * in Table 1). Cell type was confirmed by microscopy and flow cytometry as previously described [38].

The AML-derived cell lines were obtained from the DMSZ (German Collection of Microorganisms and Cell Cultures) and European Collection of Cell Cultures where they are authenticated by DNA-fingerprinting. In the laboratory they are used at low passage number for a maximum of 6 months post-resuscitation, testing regularly for Mycoplasma infection

### Virus construction and infection

MicroRNA sequence miRNA-BTK (5′-TTCACTGGACTCTTCACCTCT-3′) targeting human BTK was selected with Invitrogen Block-iT RNAi Designer software (www.invitrogen.com/rnai) and plasmid pcDNATM6.2-GW/EmGFP-miR-neg (Invitrogen) was used as source for negative control as previously described [19] [38].

### Western immunoblotting

Sodium dodecyl sulfate-polyacrylamide gel electrophoresis and Western blot analyses were performed as described previously. Briefly, whole cell lysates as well as nuclear and cytosolic were extracted and sodium dodecyl sulfate-polyacrylamide gel electrophoresis separation performed [39, 40]. Protein was transferred on nitrocellulose membrane and Western blot analysis performed with the indicated antisera according to their manufacturer’s guidelines

### Migration Assays

Migration assays were performed in triplicate in Transwell permeable plates with 8.0μM pores (Costar). The lower compartment contained 500μL of serum free media supplemented with 100 ng/ml SDF1 and the cells were applied to the upper compartment and allowed to migrate for 3 h. The amount of viable migrated cells was determined by counting using trypan blue exclusion and expressed as a percentage of the input. The bars represent the means ± SD of at least three independent experiments, each assayed in triplicate.

### Statistical analyses

Student’s T test was performed to assess statistical significance from controls. Results with P < 0.05 were considered statistically significant (*). Results represent the mean ± SD of 3 independent experiments. For Western blotting, data are representative images of 3 independent experiments.
